# Online Personalized Preference Learning Method Based on In-Formative Query for Lane Centering Control Trajectory

**DOI:** 10.3390/s23115246

**Published:** 2023-05-31

**Authors:** Wei Ran, Hui Chen, Taokai Xia, Yosuke Nishimura, Chaopeng Guo, Youyu Yin

**Affiliations:** 1 School of Automotive Studies, Tongji University, Shanghai 201804, China; ranwei@tongji.edu.cn (W.R.);; 2 JTEKT Corporation, Nara 634-8555, Japan; 3 JTEKT Research and Development Center (WUXI) Co., Ltd., Wuxi 214161, China

**Keywords:** online learning, preference learning, utility theory, Bayesian approach, LCC trajectory

## Abstract

The personalization of autonomous vehicles or advanced driver assistance systems has been a widely researched topic, with many proposals aiming to achieve human-like or driver-imitating methods. However, these approaches rely on an implicit assumption that all drivers prefer the vehicle to drive like themselves, which may not hold true for all drivers. To address this issue, this study proposes an online personalized preference learning method (OPPLM) that utilizes a pairwise comparison group preference query and the Bayesian approach. The proposed OPPLM adopts a two-layer hierarchical structure model based on utility theory to represent driver preferences on the trajectory. To improve the accuracy of learning, the uncertainty of driver query answers is modeled. In addition, informative query and greedy query selection methods are used to improve learning speed. To determine when the driver’s preferred trajectory has been found, a convergence criterion is proposed. To evaluate the effectiveness of the OPPLM, a user study is conducted to learn the driver’s preferred trajectory in the curve of the lane centering control (LCC) system. The results show that the OPPLM can converge quickly, requiring only about 11 queries on average. Moreover, it accurately learned the driver’s favorite trajectory, and the estimated utility of the driver preference model is highly consistent with the subject evaluation score.

## 1. Introduction

Advanced driver assistance systems (ADAS) such as adaptive cruise control (ACC), forward collision warning, lane keeping assistance, and lane change assistance have become increasingly common in newly manufactured vehicles. The ADAS is expected to improve driving safety and comfort. However, to be effective, ADAS systems must match drivers’ preferences and driving behaviors [1,2]. Drivers’ preferences vary based on personality traits, driving experience, and situational factors. Therefore, ADAS systems must be personalized [3,4].

Personalization methods for ADAS systems can be divided into explicit and implicit personalization approaches [3]. Explicit personalization requires drivers to manually choose a specific system setting that matches their preference. ACC is an example of explicit personalization, where drivers can set their desired speed and choose between predefined time gaps when using ACC. However, explicit personalization can be difficult for drivers to understand and may be limited in terms of the available options, particularly when the settings are interactive between multiple ADAS systems. Limited choices are another drawback of explicit personalization.

Implicit personalization is a promising approach for resolving these challenges by developing a personalized driver preference model that can predict preferences based on collected driver data. One common method for implicit personalization is driving style identification, where individual drivers are classified into driving style categories such as comfortable, normal, and sporty based on individual driving data [5,6]. In study [7], a driver style identification method was proposed based on questionnaire surveys and corresponding driving behavior characteristics. This approach was used to identify the driver type (aggressive, ordinary, or cautious) online, and a driver-adaptive ACC/CA (collision avoidance) fusion control strategy was designed accordingly. However, as highlighted in a review by [8] on the driving style identification method used for ADAS, this method still faces the challenge of limited categories and may not be able to adapt to individual preferences.

Imitation learning is another widely researched implicit personalization method, where the vehicle controller is personalized based on a model built from a group of driver behaviors [9]. The general process of this method involves observing driver behavior by collecting driving data from a group of drivers, building a driver behavior or preference model, and obtaining a personalized vehicle controller based on the driving behavior model and the measured driving data of a new individual driver [3,4]. The driver behavior model can be built based on a steering or car-following driver model [10,11] and machine learning methods [12,13,14], such as inverse reinforcement learning, which has been used to learn human-like driving [15,16,17] since the work in Ref. [18]. However, these methods assume that drivers prefer the vehicle to drive like themselves, which is not necessarily true for all drivers [19]. For instance, assertive drivers prefer a significantly more defensive driving style than their own [20,21]. Ref. [22] revealed that the perceived control of risk-taking for drivers and passengers is different. Passengers who are out of control of their vehicles perceive more risk than drivers in control of their vehicles. Therefore, personalized ADAS must consider drivers’ real preferences when designing their systems.

Preference learning is a type of machine learning that involves learning from observations that reveal information about an individual’s preferences [23]. It has been applied in various fields, including user preference mining and human–robot interaction. There are three main types of preference information used in preference learning: pairwise comparison, ranking, and rating of alternatives [24]. In human–robot interaction, learning user preferences for robot motion trajectories can be challenging due to the quality and quantity of user feedback. Learning from demonstration (LFD) is a common method used to learn user preferences [25,26], but it can be difficult for users to provide demonstrations that orchestrate all of the robot’s degrees of freedom [27]. Preference queries, such as paired-group comparison, are an easier form of user feedback, but they require a large amount of data [28]. To reduce the amount of data required, active learning or active query selection methods are used [29,30,31]. Other tricks, such as batch active preference learning [32] and scale feedback [33], are also used to further reduce the data needed. In study [34], a generative adversarial network (GAN) has been used to learn human preferences with fewer queries required. This approach replaces the role of a human in assigning preferences.

At present, great progress to learn user preference in human–robot interaction has been made. However, these works mainly focus on mobile manipulators such as personal robots and assembly line robots. Applying preference learning methods to ADAS or autonomous vehicles is a challenge as driving tasks are much more difficult to demonstrate compared to mobile manipulators, especially for unskilled drivers.

The acceptable number of queries for drivers is also much less than that for robots. Even with tricks such as batch learning, about 100 queries are required to obtain driver preference [32], which is unacceptable for drivers. Ref. [35] uses an augmenting comparison query with feature queries and an active query selection method to learn the driver’s reward function for trajectory, which is faster than preference comparison only. However, it does not provide a convergence criterion to indicate when to stop queries and obtain the final driver preference. The feature query requires drivers to carefully point out the difference in the host vehicle’s pose, relative position to the road lane, and other vehicles between paired-group trajectories, which is not easy for drivers. Therefore, there is a need for further research to develop efficient and effective preference learning methods that consider the challenges specific to ADAS and autonomous vehicles.

Based on preference learning methods, this study aims to develop an online personalized preference learning method (OPPLM) with a particular focus on the trajectory of lane centering control (LCC) in a simple curve condition without other vehicles involved. The main contributions of this paper are:

1. Introducing an online personalized preference learning method (OPPLM) based on pairwise comparison group preference queries and Bayesian approach.
2. Establishing a two-layer hierarchical structure model based on utility theory to model driver preferences on trajectory, taking into account the uncertainty of drivers’ query answers.
3. Utilizing informative and greedy query selection methods to improve the learning speed. A convergence criterion is proposed to indicate when the driver’s preferred trajectory has been found.

The paper is organized as follows: Section 2 introduces the proposed OPPLM. Section 3 describes the user study experiment conducted to validate the OPPLM. The experiment’s results are presented in Section 4, followed by a discussion in Section 5. Finally, the proposed OPPLM is summarized, and its limitations are pointed out.

## 2. Methods

### 2.1. Flow of the Online Personalized Preference Learning Method

**Figure 1:**
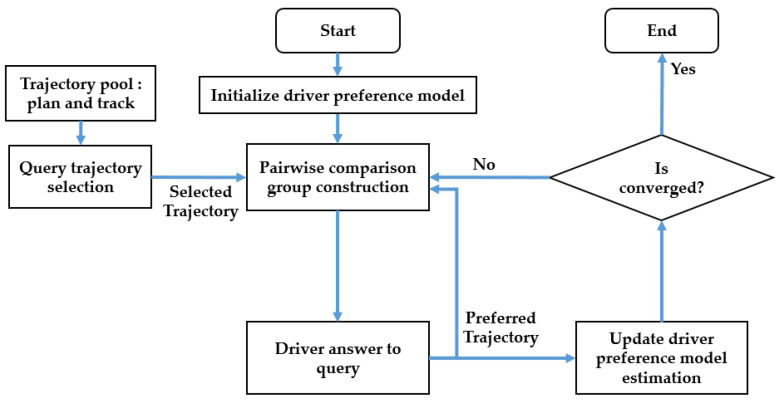
Flow chart of the OPPLM.

Figure 1 illustrates the flow chart of the proposed OPPLM. The driver preference model is initialized at the beginning and then updates online. The system starts by selecting a trajectory from the pre-prepared trajectory pool, which contains many alternative trajectories. Next, a new pairwise comparison group is constructed based on the selected trajectory and the driver’s preferred trajectory from the previous query. The driver is then queried for their preferred trajectory, and the driver preference model is updated accordingly. Finally, the OPPLM checks whether it has converged or not. If it has, the learning process ends, and the driver-preferred trajectory is identified. Otherwise, the process repeats from the first step.

### 2.2. Formulation of Driver Preference Model and Estimation Method

#### 2.2.1. Formulation of Driver Preference Model

Utility theory is widely used to model discrete choice problems. In this theory, a decision maker selects the alternative with the highest utility among those available [36]. The utility of an alternative is typically modeled as a function of its relevant attributes, often a linear function. To account for the uncertainty of the decision maker, a random utility is added to the utility function, which makes the discrete choice problem probabilistic. In this study, the driver’s preferred trajectory is modeled as the one with the highest expected utility among all alternatives, while the preferred trajectory of the pairwise comparison group is modeled as the one with a higher expected utility.

The relevant attributes of trajectory for vehicles typically include safety, comfort, efficiency, and energy-saving. However, for simplicity, energy-saving is not considered in this study. Let $U_{S}$, $U_{C}$, and $U_{E}$ represent the safety, comfort, and efficiency utility, respectively. Let $\beta_{S}$, $\beta_{C}$, and $\beta_{E}$ represent the linear weight parameters of the safety, comfort, and efficiency utility, with ε representing the random utility. Therefore, the utility (*U*) of a trajectory could be represented as follows:

(1) $$U=\beta_{S}\cdot U_{S}+\beta_{C}\cdot U_{C}+\beta_{E}\cdot U_{E}+\varepsilon =\beta \Theta^{T}X+\varepsilon$$

where, $Χ={\left(U_{S},\;U_{C},\;U_{E}\right)}^{T}$, ${\left(\beta_{S},\;\beta_{C},\;\beta_{E}\right)}^{T}=\beta {\left(\theta_{S},\;\theta_{C},\;\theta_{E}\right)}^{T}=\beta \Theta^{T}$. $\theta_{S},\;\theta_{C}$, and $\theta_{E}$ represent the normalized linear weight parameters of the safety, comfort, and efficiency utilities, respectively, all within [0, 1]. $\beta =|\beta_{S}|+|\beta_{C}|+|\beta_{E}|$ represents the normalization coefficient.

The safety, comfort, and efficiency utility $\left(U_{S},\;U_{C},\;U_{E}\right)$ of a trajectory are unknown. However, they can be calculated using assumed utility functions and corresponding trajectory attributes or characteristic indicators. Let $X_{S\_1},\;X_{S\_2},\ldots$ represent the safety corresponding trajectory indicators. Let $\beta_{S\_1},\;\beta_{S\_2},\ldots$ represent the linear weight parameters of the safety, comfort, and efficiency utility. Similarly, the safety utility ($U_{S}$) can be calculated using the following equation:

(2) $$U_{S}=\beta_{S\_1}\cdot X_{S\_1}+\beta_{S\_2}\cdot X_{S\_2}+\ldots +\varepsilon =\beta_{S}{\theta_{S}}^{T}X_{S}+\varepsilon_{S}$$

where, $X_{S}={\left(X_{S\_1},X_{S\_2},\ldots \right)}^{T}$, ${\left(\beta_{S\_1},\;\beta_{S\_2},\;\ldots \right)}^{T}={\beta_{S}\left(\theta_{S\_1},\;\theta_{S\_2},\;\ldots \right)}^{T}=\beta_{S}\Theta_{S}$. The comfort and efficiency utility functions are similar but use different trajectory characteristic indicators.

The safety utility is calculated based on trajectory indicators. It means that the safety utility is the driver’s direct perception of the trajectory. Therefore, it is called the safety perception model (SPM) in this research. Similarly, the comfort perception model (CPM) and efficiency perception model (EPM) are used for calculating the comfort and efficiency utility functions, respectively. The utility function of a trajectory, as shown in Equation (1), is indirectly evaluated using the safety, comfort, and efficiency utility functions, and it is referred to as the utility evaluation model (UEM).

For pairwise trajectory comparison group (*A*, *B*), the probability that a driver with utility function parameter (β,Θ) prefers trajectory *A* to *B*, represented by $P_{r}\left(A|\beta ,\Theta ,X_{A},X_{B}\right)$, can be modeled as the probability that the utility of trajectory *A* ($U_{A})$ is larger than that of trajectory *B* ($U_{B})$, represented by $P_{r}\left(U_{A}>U_{B}\right)$, which is formulated as:

(3) $$P_{r}\left(A|\beta ,\Theta ,X_{A},X_{B}\right)=P_{r}\left(U_{A}>U_{B}\right)=P_{r}(\epsilon_{B}-\epsilon_{A}<{\beta \Theta }^{T}{(X}_{A}-X_{B}))$$

where, $\epsilon_{A}$ and $\epsilon_{B}$ could be assumed to be an independent and identical distribution, although the specific distribution is unknown. A reasonable assumption for the distribution of ε is a Gaussian distribution, given the central limit theorem. However, this assumption does not lead to a closed-form solution for the probability. A better assumption for the distribution is the standard Gumbel (or type I extreme value) distribution, which does lead to a closed-form solution [36] as follows:

(4) $$P_{r}\left(A|\beta ,\Theta ,X_{A},X_{B}\right)=\frac{1}{1+e^{-{\beta \Theta }^{T}{(X}_{A}-X_{B})}}$$

Equation (4) models the likelihood that the driver prefers trajectory *A* to *B* for the pairwise comparison group (*A*, *B*). The probability that the driver prefers trajectory *B* to trajectory *A* can be modeled as follows:

(5) $$P_{r}\left(B|\beta ,\Theta ,X_{A},X_{B}\right)=1-P_{r}\left(A|\beta ,\Theta ,X_{A},X_{B}\right)=\frac{e^{-{\beta \Theta }^{T}{(X}_{A}-X_{B})}}{1+e^{-{\beta \Theta }^{T}{(X}_{A}-X_{B})}}$$

Based on the above equation, it is easy to predict the driver’s answer to a query. If $P_{r}\left(A|\beta ,\Theta ,X_{A},X_{B}\right)$ is larger than 0.5, then the driver is more likely to prefer *A* than *B*, and vice versa.

Equations (3)–(5) do not consider the uncertainty of a driver’s answer when distinguishing between two trajectories. Sometimes, drivers may find it difficult to discern the difference between two trajectories, and forcing them to make a deterministic choice may be inappropriate. In such cases, it is more appropriate to allow for uncertain answers. It is assumed that when the absolute difference in utility is closer to zero, the driver is more likely to give an uncertain answer. Thus, the probability of different answers to a query can be modeled as follows:

(6) $$Answer\;to\;query=\left\{\begin{array}{c} A,\;\;P_{r}\left(A|\beta ,\Theta ,X_{A},X_{B}\right)>UB\\ B,\;\;P_{r}\left(A|\beta ,\Theta ,X_{A},X_{B}\right)<LB\\ Uncertain\;\;otherwise \end{array}\right.$$

where, the *UB* (upper bound) and *LB* (lower bound) represent the probability threshold between the uncertain result and the other two deterministic results. 

To calculate the likelihood of a deterministic answer, we can use Equations (4) and (5), respectively. However, to calculate the likelihood of an uncertain answer, we can view it as a joint result of two opposite answers:

(7) $$P_{r}\left(A\approx B|\beta ,\Theta ,X_{A},X_{B}\right)=P_{r}\left(A|\beta ,\Theta ,X_{A},X_{B}\right)\cdot P_{r}\left(B|\beta ,\Theta ,X_{A},X_{B}\right)=\frac{e^{-\beta \Theta^{T}{(X}_{A}-X_{B})}}{{\left(1+e^{-\beta \Theta^{T}{(X}_{A}-X_{B})}\right)}^{2}}$$

where, $P_{r}\left(A\approx B|\beta ,\Theta ,X_{A},X_{B}\right)$ represents the probability that the driver’s answer is uncertain.

**Figure 2:**
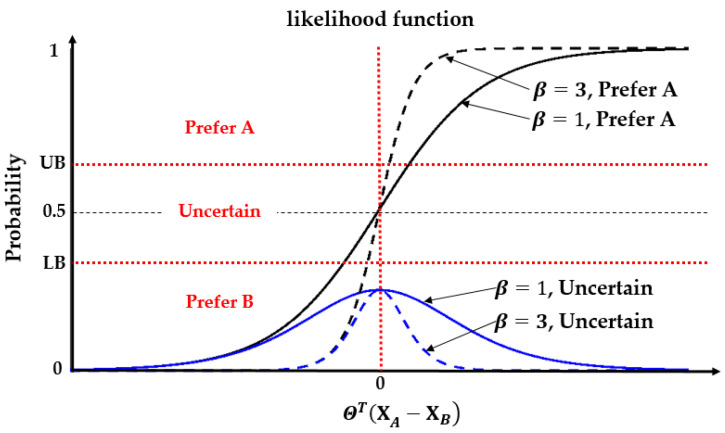
The correlation between the likelihood of different answers to a query and the utility difference $\Theta^{T}{(X}_{A}-X_{B})$ for drivers with different parameters β.

The correlation between the likelihood of different answers to a query and the utility difference $\Theta^{T}{(X}_{A}-X_{B})$ for drivers with different parameters β is shown in Figure 2.

Figure 2 illustrates that, on one hand, the likelihood that the driver prefers A to B increases as the utility difference $\Theta^{T}{(X}_{A}-X_{B})$ becomes larger. However, as $\Theta^{T}{(X}_{A}-X_{B})$ approaches 0, the likelihood of an uncertain answer increases. On the other hand, for the same utility difference $\Theta^{T}{(X}_{A}-X_{B})$, the likelihood of uncertainty decreases as the parameter *β* increases, which means that drivers are more likely to give a deterministic answer to a query. The parameter β measures the driver’s ability to distinguish between trajectories and is therefore referred to as the perception coefficient in this study. It is worth noting that, for a specific critical utility difference, e.g., the minimum difference required for a driver to give a deterministic answer, the perception coefficient *β* and the corresponding probability thresholds *UB* and *LB* are interrelated. As the perception coefficient β increases, the values of *UB* and *LB* approach 0.5. This coupling indicates that the parameters *UB*, *LB*, and β are interdependent.

Overall, the personalized preference learning system aims to estimate the linear weight parameters Θ and the perception coefficient β of the driver preference model (UEM, SPM, CPM, EPM) for each individual.

#### 2.2.2. Estimation Method

**Figure 3:**
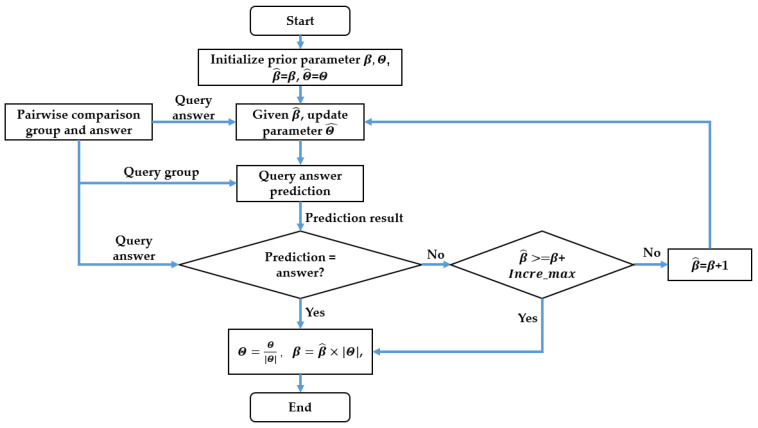
The flow chart of the parameters update method for query results at each step.

The driver preference model parameters are estimated using a Bayesian approach and a limited greedy estimation method. Firstly, an assumption is made about the prior probability distribution of estimation parameters. Then, the estimation is updated based on the driver’s preference trajectory query result of a pairwise trajectory comparison group (*A*, *B*) at every step, following the Bayesian approach. The flow chart of the parameter update method for query results at each step is shown in Figure 3.

The first step of the estimation process involves updating the parameter for a given prior parameter set Θ and perception coefficient β. For the pairwise comparison group (*A*, *B*) and its corresponding driver query answer, the parameter Θ can be updated using the following equation:

(8) $$P_{r}\left(\Theta |\beta ,Ans,X_{A},X_{B}\right)=P_{r}\left(\Theta \right)\cdot P_{r}\left(Ans|\beta ,\Theta ,X_{A},X_{B}\right)$$

where, $P_{r}\left(\Theta \right)$ represents the prior probability distribution of estimation parameters Θ, and $P_{r}\left(Ans|\beta ,\Theta ,X_{A},X_{B}\right)$ represents the likelihood of the query answer as calculated by Equations (4)–(7). $P_{r}\left(\Theta |\beta ,Ans,X_{A},X_{B}\right)$ represents the posterior probability distribution of the updated Θ.

The second step of the estimation process involves determining whether the updated driver preference model, with its newly estimated parameters, can correctly predict the latest query result. The prediction of the latest query result can be calculated using Equation (6). If the prediction matches the real driver answer, then the parameter estimation update ends, and the perception coefficient β remains unchanged. However, if the prediction does not match the real driver answer, the third step involves incrementally increasing the perception coefficient β and repeating the first and second steps until the prediction is consistent with the driver answer. This process is referred to as “greedy” because the likelihood of the answer increases with β, as shown in Figure 2, leading to a more accurate prediction by the driver preference model with updated parameters. To ensure parameter estimation stability and prevent noisy answers from significantly decreasing estimation accuracy, the increment of β is limited to a value no larger than a specified number Incre_max for each query answer. This is why the estimation method is referred to as “limited-greedy”. Finally, the parameters Θ are normalized, and the perception coefficient β is modified accordingly.

### 2.3. Pairwise Comparison Group Construction and Query Trajectory Selection

To achieve accurate and efficient learning of the driver-preferred trajectory, it is crucial to construct an appropriate pairwise comparison group that minimizes the required amount of data. However, there is often a trade-off between speed and accuracy. Selecting a comparison group that leads to faster learning may result in a suboptimal outcome, while a more accurate approach may require more data and time. This is similar to the exploration–exploitation dilemma encountered in reinforcement learning, which can be addressed using the ϵ-greedy method [37]. In reinforcement learning, exploitation refers to selecting actions that have the highest expected reward based on current experience, while exploration involves selecting untried actions in the hopes of achieving a higher reward.

Drawing on the ϵ-greedy method, we propose a similar "greedy" policy to construct the pairwise comparison group. This involves combining a new query trajectory with one that has been previously compared, at each step, to minimize the required number of query trajectories. The greedy approach is reflected in both the construction of the pairwise comparison group and the selection of new query trajectories.

#### 2.3.1. Pairwise Comparison Group Construction

The greedy pairwise comparison group is constructed by selecting the two trajectories that the driver is most likely to prefer. The first trajectory is the driver’s favorite among all compared trajectories, representing the greediest selection among them. A second trajectory selection method is introduced below.

#### 2.3.2. Query Trajectory Selection

A greedy query trajectory selection policy entails selecting the trajectory that the driver is most likely to prefer according to the current driver preference model, except for the first one selected above. On the other hand, the ϵ-greedy policy involves selecting a greedy trajectory with probability 1−ϵ at each step, while all non-greedy trajectories are selected at random with probability ϵ, the exploration probability, as expressed in Equation (9):

(9) $$Pr\_selected\left(Trajectory\right)=\left\{\begin{array}{c} 1-\epsilon +\frac{\epsilon }{N},\;if\;Trajectory\;is\;greedy\\ \frac{\epsilon }{N},\;\;otherwise \end{array}\right.$$

**Figure 4:**
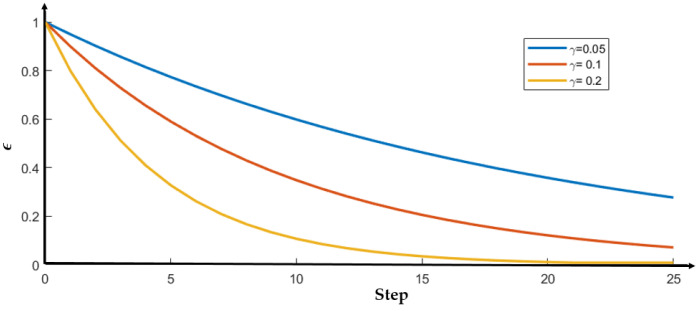
The exploration probability ϵ decreases as the step increases, and it decreases faster with a larger decay rate γ.

Here, $Pr\_selected\left(Trajectory\right)$ represents the probability of the trajectory being selected, and *N* is the number of all alternative trajectories. This equation ensures that each trajectory has a chance of being selected. As the value of ϵ increases, the probability of selecting the greedy trajectory decreases, while the other trajectories become more likely to be selected, thus increasing the level of exploration. To increase the level of exploration at the start and decrease it after some queries to speed up convergence, a variable exploration level is proposed as given in Equation (10):

(10) $$\epsilon =\epsilon_{ini}\cdot {\left(1-\gamma \right)}^{step}$$

where, $\epsilon_{ini}$ is the initial value, and γ is the decay rate. Figure 4 illustrates how ϵ changes as the step increases, with an initial value of $\epsilon_{ini}=1$ and three different values of decay rate γ. The figure shows that as γ increases, ϵ decreases faster, leading to a decrease in the level of exploration.

### 2.4. Convergence Criterion

**Figure 5:**
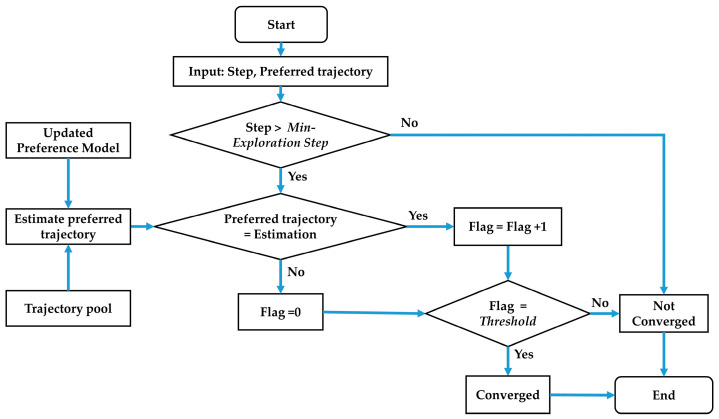
Flow chart of convergence criterion.

A convergence criterion is proposed to end preference learning as shown in Figure 5 below.

Firstly, it is required to check whether the current step is larger than the Min-Exploration Step, which guarantees a minimum number of queries. The second step is to match up the current driver preferred trajectory with the preference estimation, which is the trajectory with the highest utility among all trajectories in the trajectory pool. In addition, the trajectory utility can be calculated using Equation (1) with the newly updated preference model parameters. If they match, the Flag representing the number of consecutive false comparisons should be reset to zero. However, if the comparison is true, the Flag should be incremented by one. The final step is to judge whether the Flag is equal to the Threshold to ensure a stable preference estimation. This criterion should not be too conservative, resulting in numerous inefficient comparison queries, nor too aggressive, resulting in premature termination and inaccurate estimation. 

## 3. Experiment Configuration

The proposed preference learning method (OPPLM) was evaluated through a user study conducted on a fixed-base driving simulator.

### 3.1. Equipment

**Figure 6:**
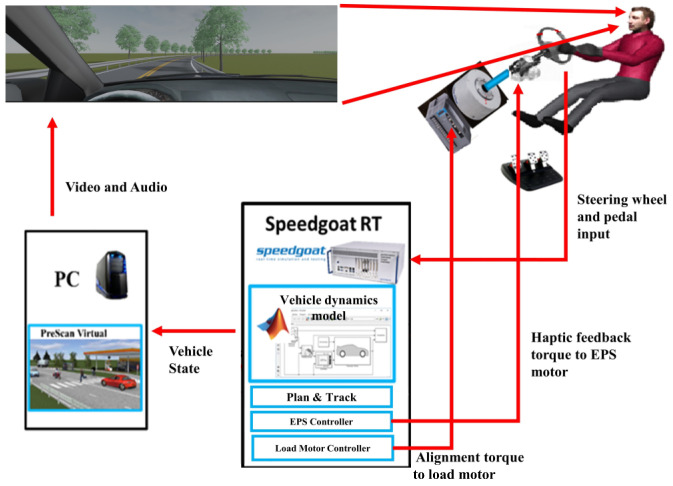
Configuration of experiment equipment.

The fixed-base driving simulator comprises four components, as shown in Figure 6. These include a real-time target machine, a steering system and pedals, a personal computer, and a screen. The real-time target machine is responsible for computing trajectory planning, tracking, and controlling the steering system and pedals. Specifically, it sends alignment torque to the load motor and haptic feedback torque to the EPS (Electric Power Steering) motor and outputs the vehicle state to the scenario simulation software (prescan) running on the personal computer. The real-time scenario is then displayed on a screen with a resolution of 3840 × 1080.

### 3.2. Scenario

**Figure 7:**
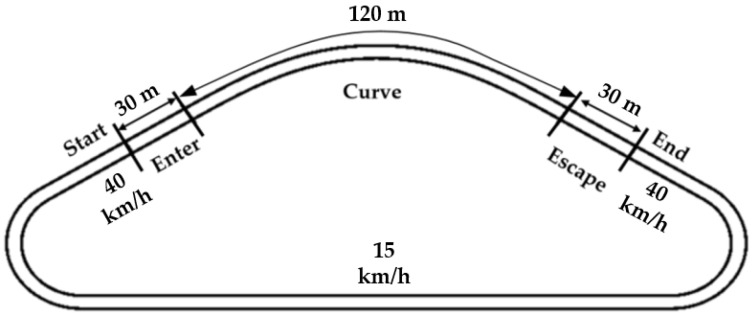
Designed closed-loop test scenario.

The experiment was conducted in a simple single-lane curve scenario without any other vehicles present. To speed up the experiment procedure, a closed-loop triangular field was designed, as shown in Figure 7.

Throughout the entire experiment, the vehicle was controlled by a controller, and the test subject did not need to drive manually. The vehicle initially entered the curved road at a speed of 40 km/h and exited the curve with an end speed of 40 km/h. Within the curve, the vehicle was controlled to follow the planned trajectory, and, outside the curve, it traveled at a constant speed of 15 km/h on the long straight road to allow the subjects enough time to evaluate. Smooth speed profiles connected the speed profiles between these sections to avoid discomfort.

### 3.3. Trajectory Pool

To obtain a driver-preferred trajectory under curved driving conditions, a trajectory planning method capable of generating diverse trajectories is introduced. Additionally, the trajectory track method utilized in this research is presented. The trajectory pool is established based on the planning and tracking methods employed in the study.

#### 3.3.1. Trajectory Planning

**Figure 8:**
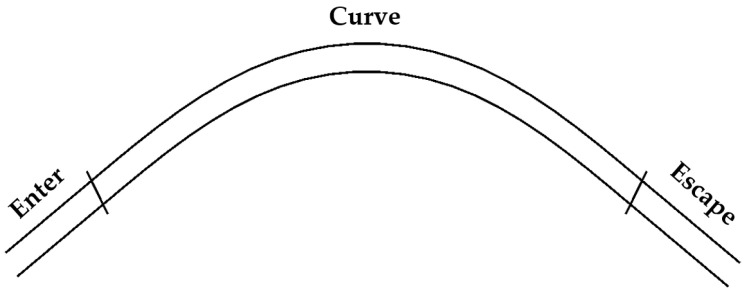
Three-curve road segments.

The trajectory of the curved path is composed of three distinct segments, each corresponding to a particular section of the road, as depicted in Figure 8.

Referring to the modes established in Ref. [38], path planning is achieved by combining various modes with different weights to generate divergent paths. For the entry and exit segments, the path is planned by connecting the endpoint of the middle segment of the curve to the center of the road lane using a cubic spline.

Speed planning is also conducted using the optimal speed planning methodology outlined in Ref. [38], with certain modifications to adapt to the curve conditions. Specifically, the minimum jerk mode, which minimizes the derivative of longitudinal acceleration, is substituted with the minimum speed variation mode, which aims to minimize variations in longitudinal speed. In addition, the maximum allowable velocity is replaced by the maximum allowable lateral acceleration, which is used to constrain the maximum speed during the curve. For further details, please refer to Ref. [38].

**Figure 9:**
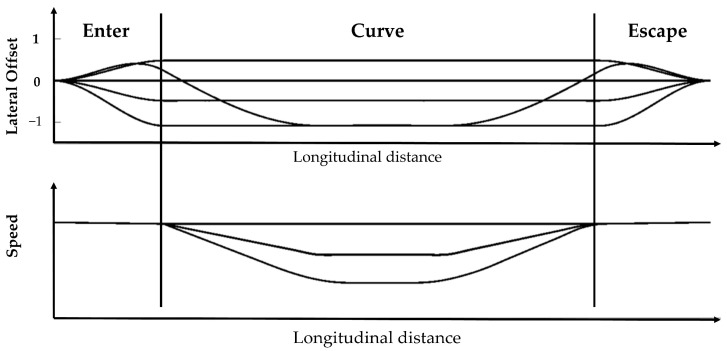
The planned paths and speed profiles in curve with different parameters.

Figure 9 illustrates some of the planned paths and speed profiles presented in the Frenet coordinate system where a negative lateral offset indicates positions located close to the inner side of the curved road, and a positive lateral offset indicates positions close to the outer side. The lateral offsets −1, 0, and 1 indicate the inner side, center, and outer side of the road, respectively. The speed profile consists of three constant speed profiles, which are smoothly connected. The minimum constant speed is determined by the maximum allowable lateral acceleration.

#### 3.3.2. Trajectory Tracking

The augmented Stanley controller [39] is utilized to track the various planned trajectories. The tracking error is typically less than 5 cm when following the planned trajectories, with a maximum error of less than 10 cm. This level of tracking performance is deemed acceptable and ensures that the distinct planned trajectories can be clearly discerned by the driver.

#### 3.3.3. Trajectory Pool Setting

A trajectory pool consisting of thirty unique trajectories is generated by manipulating various parameters of the trajectory planning method. These thirty trajectories are obtained through an orthogonal design of ten paths and three distinct speed profiles. The speed profiles differ mainly based on the minimum speed required during the curve, which is set to 20 km/h, 30 km/h, and 40 km/h.

### 3.4. Driver Preference Model and Estimation Method Setting

#### 3.4.1. Trajectory Indicators

In line with similar research, trajectory indicators utilized to construct the SPM, CPM, and EPM were selected based on prior experience. The trajectory indicators commonly used to identify driving styles [8] and personalize ADAS [4] (p. 400) were reviewed, including speed, acceleration, jerk, and distance to the lane center, among others. From this review, three trajectory indicators were selected for each perception model, as detailed in Table 1 below.

#### 3.4.2. Estimation Method Setting

The parameters of the preference model were estimated without prior knowledge by assuming a normal distribution with a mean of (1/3, 1/3, 1/3) and an identity covariance matrix for the prior probability distribution of estimation parameters Θ. The initial perception coefficient was assumed to be one, and the increment limitation Incre_max of the β was set to three. In order to determine convergence, the minimum number of queries Min-Exploration was set to four, while the Threshold, which ensures a stable preference estimation, was set to three.

### 3.5. Subjects

In order to ensure an effective statistical analysis with a sufficient number of subject samples, the G*power software [40] was utilized to determine the minimum required sample size. A paired-samples t-test was employed to validate the results, with an effect size set at 0.5, an α error probability of 0.05, and a power of 0.8. As a result, a total sample size of 27 was determined. In the experiment, 29 subjects ultimately participated, with an average age of 34.9 (SD = 11.8). Among the subjects, 15 were identified as inexperienced drivers with an average annual driving mileage of less than 1000 km, while 14 were identified as experienced drivers with an average annual driving mileage of more than 20,000 km.

### 3.6. Procedure

Before conducting the main experiment, a pre-comparison test was conducted to ensure that each subject was familiar with the scenario and experiment procedure. Four pairwise comparison groups were presented to each subject, and they were required to experience the trajectories in the driving simulator, followed by describing the differences between the trajectories. The subjects were allowed to experience each trajectory multiple times until they were confident with the comparison. In case of any ignored differences, they were informed and the trajectories were re-compared. After comparing all four groups, the subjects were required to provide answers to the four queries listed in Table 2.

During the experiment, each participant was instructed to respond to the four queries listed in Table 2 for every comparison group. The selection of query trajectories and the construction of comparison groups were both automated using the method described in Section 2.3. The first comparison group required the participant to answer the queries after two trajectories were presented in turn. Subsequently, the participant was required to compare the newly selected trajectory to the preferred one in the last query and repeat the process until the OPPLM converged.

Once the OPPLM converged, four trajectories were selected to validate the effectiveness of the learned driver preference model. The utility of each trajectory in the pool of 30 trajectories was calculated using Equations (1) and (2) with the newly estimated driver preference model parameters. The 30 trajectories were then sorted in descending order of utility, and the leading two trajectories, the 15th (middle) trajectory, and the last trajectory according to their utility were selected for evaluation using the Likert scale presented in Table 3.

### 3.7. Evaluation Indices

To evaluate the effectiveness of the OPPLM, two aspects are considered: learning speed and accuracy. The learning speed is measured by the number of queries (*QN*) required for the OPPLM to converge. The learning accuracy is evaluated by two indices. The first index is the goodness-of-fit (*GOF*) of the final learned driver preference model, which is calculated by the ratio of *QN_Positive* to *QN*, as given by Equation (11).

(11) $$GOF=\frac{QN\_Positive}{QN}$$

Here, *QN_Positive* refers to the number of queries that can be correctly predicted by Equation (6) with the learned preference model parameters. In addition, *QN* refers to the queries number that is used when the OPPLM converges. A higher value of GOF indicates greater accuracy of the model.

The second index is the score–utility consistency (*SUC*), which measures the consistency between the evaluation Likert score ordering and the corresponding utility ordering for the final evaluated four trajectories. Taking the item preference (the fourth item in Table 3) as an example. Let *A*, *B*, *C*, and *D* represent the four trajectories that are selected to be evaluated after the OPPLM converges in Section 3.6. They are sorted by utility in descending order, meaning that the utility of the four trajectories is ranked as $U_{A}>U_{B}>U_{C}>U_{D}$. Let *a*, *b*, *c*, and *d* represent the preference evaluation score of the corresponding four trajectories. The *SUC* for item preference could be calculated as follows:

(12) $$SUC=\frac{sign\left(a-b\right)+sign\left(b-c\right)+sign\left(c-d\right)}{3}$$

Here, *sign*() is the sign function. *Sign*(*a*−*b*) equals 1 when (*a*−*b*) is positive; sign(*a*−*b*) equals 0 when (*a*−*b*) equals 0; and *sign*(*a*−*b*) equals -1 when (*a*−*b*) is negative. For example, if the evaluation scores of the four trajectories are *a* = 6, *b* = 5, *c* = 4, and *d* = 3, the evaluation score ordering is consistent with the utility ordering, indicating that the learned utility model accurately predicts the driver’s preference. In this case, the *SUC* is equal to 1. Conversely, if the scores of the four trajectories are *a* = 3, *b* = 4, *c* = 5, and *d* = 6, the estimated utility is opposite to the driver’s preference for trajectory, resulting in an *SUC* of −1. The *SUC* is within the range of [−1, 1]. The *SUC* value provides an indication of the accuracy of the model, with a higher *SUC* indicating greater accuracy.

## 4. Results

This section presents an analysis of the learning speed and accuracy of the experiment results.

### 4.1. Learning Speed

**Figure 10:**
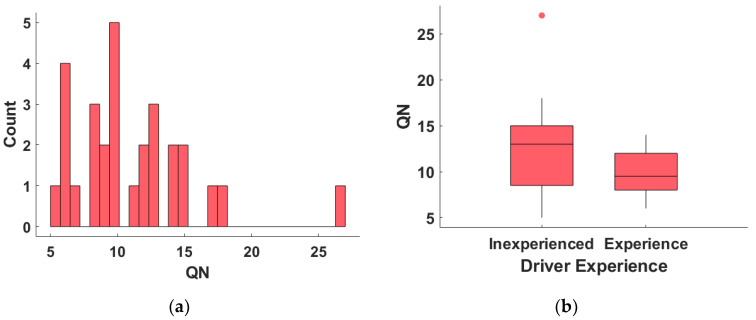
The result of *QN* for all subjects: (**a**) the frequency distribution of *QN* for all subjects; (**b**) the boxplot of *QN* for inexperienced and experienced subjects.

The distribution of the number of queries (*QN*) of all 29 subjects is presented in Figure 10.

The mean *QN* of all participants is 11.1 with a standard deviation of 4.6. The vast majority of subjects (excluding one outlier) had a *QN* of less than 20, indicating that the OPPLM generally converged within 20 queries. Notably, one participant had a *QN* of 27, which deviates significantly from the remaining results. In Figure 10b, the *QN* of inexperienced and experienced drivers is compared. The mean *QN* of the inexperienced subjects is 12.4, with a standard deviation of 5.7. In comparison, the mean *QN* of experienced subjects is 9.7, with a standard deviation of 2.6. An independent-samples t-test was conducted to examine the *QN* difference between inexperienced and experienced subjects. The results indicate that the influence of subject experience on *QN* is not statistically significant (t(27) = 1.61, *p* = 0.12).

### 4.2. Learning Accuracy

#### 4.2.1. Goodness-of-Fit

**Figure 11:**
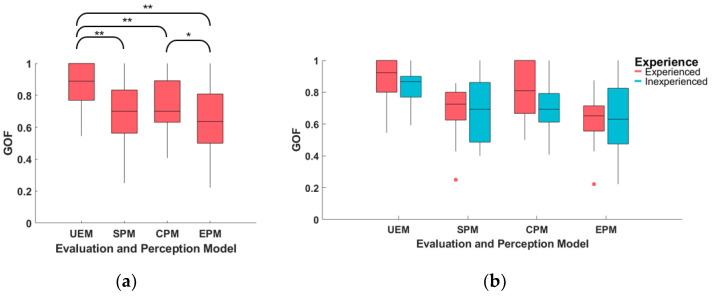
The goodness-of-fit (GOF) of the driver preference model (UEM, SPM, CPM, and EPM) for all subjects. (**a**) Boxplot of different models; (**b**) boxplot of different models for experienced and inexperienced subjects. ** means the statistical test p<0.01, * means p<0.05.

This section presents an analysis of the goodness-of-fit (GOF) of the four driver preference models (UEM, SPM, CPM, and EPM) for 29 subjects, as shown in Figure 11 and Table 4.

The UEM model had a mean GOF of 0.85 across all subjects, indicating that 85% of the queries could be correctly predicted by the learned UEM. This result suggests that the UEM performs well in modeling subject preference queries in a pairwise comparison group. The GOF for the other three perception models (SPM, CPM, and EPM) were all above 0.64. To compare the performance of each model, a paired-sample t-test was conducted between each two of the four models, as depicted in Figure 11a. The results indicate that the GOF of UEM is significantly higher than that of SPM (t(28) = 3.98, *p* = 0.000), CPM (t(28) = 3.25, *p* = 0.003) and EPM (t(28) = 4.78, *p* = 0.000). Additionally, the GOF of CPM was found to be significantly larger than that of EPM (t(28) = 4.78, *p* = 0.016). 

Moreover, the influence of subject experience on the GOF of the models was investigated. The results demonstrate that the mean GOF of the experienced subjects was higher than that of the inexperienced subjects for all models. However, an independent-samples t-test revealed no significant difference between experienced and inexperienced subjects for each model (UEM: t(27) = 1.04, *p* = 0.308; SPM: t(27) = 0.12, *p* = 0.905; CPM: t(27) = 1.56, *p* = 0.130; EPM: t(27) = 0.17, *p* = 0.861).

#### 4.2.2. Score–Utility Consistency

**Figure 12:**
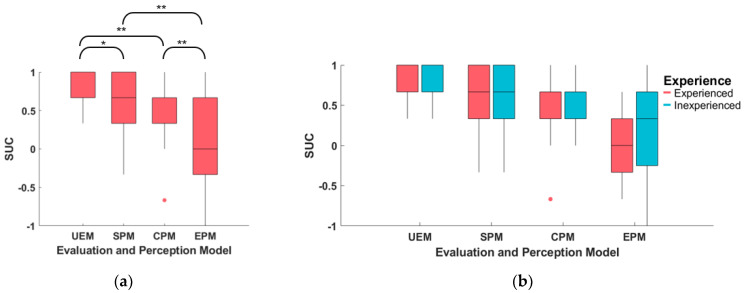
The score–utility–consistency (SUC) of the driver preference model (UEM, SPM, CPM, and EPM) for all subjects. (**a**) Boxplot of different models; (**b**) boxplot of different models for experienced and inexperienced subjects. ** means the statistical test p<0.01, * means p<0.05.

The score–utility–consistency (SUC) of the driver preference model (UEM, SPM, CPM, and EPM) of 29 subjects is summarized and presented in Figure 12 and Table 5.

The mean SUC of UEM for all subjects is 0.74, indicating good consistency between the order of estimated utility and the evaluation score. This suggests that UEM models the degree of the subjects’ trajectory preferences well. The SUC of the other three perception models (SPM, CPM, and EPM) is smaller than that of UEM, with EPM having the smallest SUC value. Paired-sample t-tests are conducted for each pair of models, and the results are displayed in Figure 12a. The analysis reveals that the SUC of UEM is significantly larger than SPM (t(28) = 2.24, *p* = 0.03), CPM (t(28) = 3.45, *p* = 0.002), and EPM (t(28) = 6.11, *p* = 0.000). Furthermore, the SUC of EPM is significantly smaller than that of SPM (t(28) = −3.06, *p* = 0.005) and CPM (t(28) = −2.81, *p* = 0.009).

The impact of subject experience on the SUC of the models is also studied. The results indicate that the mean SUC of experienced subjects is larger than that of inexperienced subjects for each model. However, an independent-samples t-test reveals no significant difference between experienced and inexperienced subjects for each model (UEM (t(27) = 0.052, *p* = 0.96), SPM (t(27) = 0.78, *p* = 0.442), CPM (t(27) = 0.43, *p* = 0.671), EPM (t(27) = 0.68, *p* = 0.505)).

#### 4.2.3. Evaluation Score

**Figure 13:**
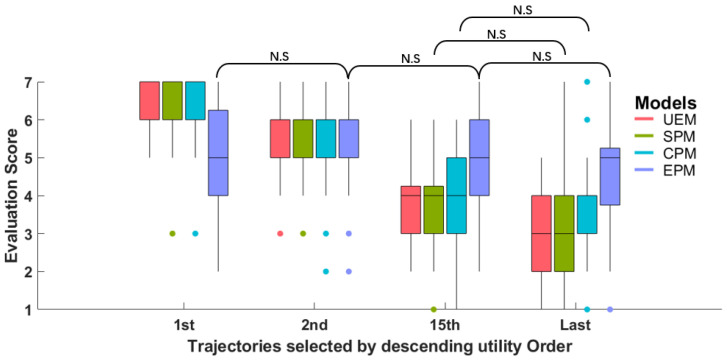
Boxplot of the evaluation scores of the selected four trajectories according to the estimated utility by UEM. N.S means statistical test p>0.05, which is not significant. The other test results are significant.

The evaluation score of the four selected trajectories, based on the estimated utility by UEM for all subjects, is presented in Figure 13. Table 6 provides a summary of the evaluation scores for UEM and the other three perception models (SPM, CPM, and EPM).

The mean score of the trajectory ranked first, by estimated utility in descending order, is 6.48, which is larger than that of the other three trajectories. A paired-sample t-test was conducted to validate the evaluation score difference between adjacent trajectory groups for the UEM model, and the result showed that the evaluation score was significantly different between adjacent groups. This qualitative result indicates that the estimated utility by UEM is consistent with subjects’ degree of preference for trajectory. The summary and statistical test results for the other three perception models are also listed in Table 6. The result shows that the estimated utility by SPM and CPM is consistent with subjects’ corresponding evaluation of safety and comfort, respectively. However, for EPM, the evaluation score of trajectories between groups has no significant difference. This indicates that the EPM could not model subjects’ evaluation of efficiency well. The qualitative result is consistent with the quantitative result of GOF and SUC, in which the GOF and SUC of the EPM are the smallest among the models.

**Figure 14:**
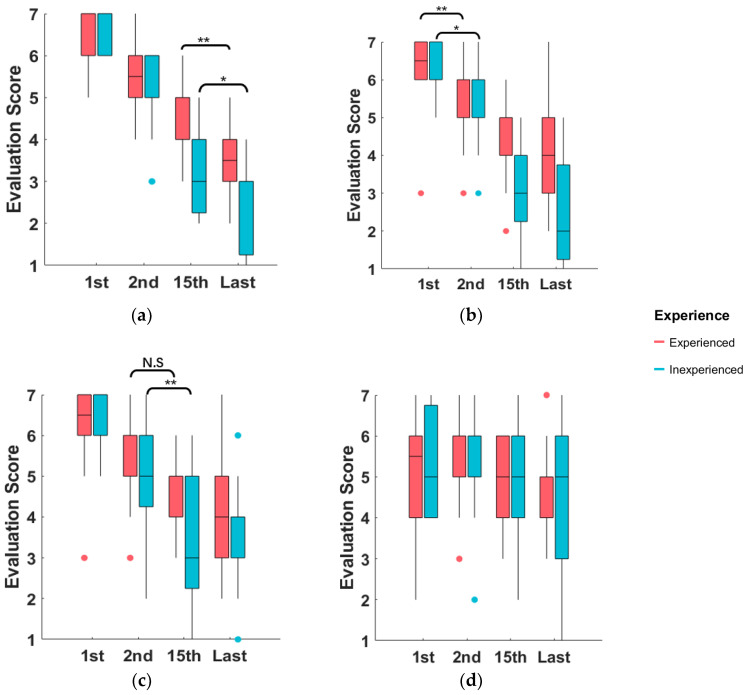
Boxplot of the evaluation scores of the selected four trajectories according to the estimated utility by different models: (**a**) UEM, (**b**) SPM, (**c**) CPM, and (**d**) EPM. The test result difference for the experienced and inexperienced driver groups are marked in the figure, in which N.S. means statistical test p>0.05, which is not significant, ** means the statistical test p<0.01, and * means p<0.05.

The influence of driver experience on the result is presented in Figure 14. A paired-sample t-test is conducted to compare the result of different driver experience groups for each model. The result shows that there is little difference between drivers with different experience, except for the CPM. For inexperienced drivers, there is a significant difference between the evaluation scores of comfort of the 2nd and 15th trajectories, but not for experienced drivers. The degree to which comfort affects drivers’ preference for trajectory is inconsistent among drivers of different experience.

## 5. Discussion

### 5.1. Learning Speed

The OPPLM converged in approximately 11 queries on average. This is significantly fewer queries than those required in human–robot-interaction related research, where as many as 100 queries are often required [32]. However, there was one subject who required 27 queries, as shown in Figure 10a, which is significantly more than the others. The reason for this is that this subject provided conflicting answers to some pairwise comparison groups. It is challenging for subjects to avoid such cases completely, and a more efficient approach is required to handle such cases and improve the convergence speed of OPPLM in the future.

Another problem with the learning speed is that OPPLM converged too early for some subjects. Six subjects’ OPPLM converged within seven queries, which occurred when the subject’s favorite trajectory was selected by accident, and the greedy trajectories were selected at the first few queries. Early convergence can result in a sub-optimal solution for finding the subject’s favorite trajectory. To avoid such cases and guarantee enough exploration, further study is needed.

In this research, the convergence and stopping of OPPLM were based on the proposed convergence criterion. However, for practical applications, convergence of OPPLM is not necessary. As long as the driver is satisfied with the current trajectory and does not request another query actively, the OPPLM will not be updated. Thus, the learning speed of a satisfied trajectory may be faster in actual application.

### 5.2. Learning Accuracy

The mean goodness of fit (GOF) for UEM, calculated within the range of 0–1 for all subjects, is 0.85, while the mean score–utility–consistency (SUC), calculated within the range of [−1, 1], is 0.74. A paired-sample t-test confirms a significant difference in the evaluation score between two adjacent trajectory groups for UEM, indicating that UEM models the subject’s degree of preference for trajectory well. However, for some subjects, the GOF of UEM is less than 0.6, and the SUC is less than 0.4. Nonetheless, the accuracy of UEM is considerably higher than that of the other three perception models (i.e., SPM, CPM, and EPM). This suggests that the three perception models are not estimated well, particularly EPM. The low accuracy of these models could be attributed to the direct relationship between the query trajectory selection and convergence criteria with UEM. Therefore, to enhance the learning accuracy of UEM, it is necessary to improve the estimation accuracy of the perception models.

### 5.3. Driver Preference Model Assumption and Setting

In this research, a two-layer hierarchical structure model based on utility theory is assumed to be the driver preference model. The corresponding trajectory indicators for each of the three perception models are selected based on experience. However, the selection method of indicators could be further optimized or even adapted to individual drivers to improve the accuracy of the driver preference model. Additionally, the estimation method settings in Section 3.4.2 could be further studied to improve the performance of OPPLM.

Moreover, the proposed OPPLM could be applied to other advanced driver assistance system functions or autonomous vehicles to learn drivers’ preferences based on the driver preference model. However, for different functions, the driver preference model needs to be specifically developed. Further research is needed to explore the potential applications of OPPLM in other contexts and to improve its performance.

## 6. Conclusions

This research introduced an online personalized preference learning method (OPPLM) based on pairwise comparison group preference queries and the Bayesian approach. The driver preference model was established using a two-layer hierarchical structure model based on utility theory. To improve accuracy, the uncertainty of drivers’ query answers was modeled, and informative and greedy query selection methods were used to enhance learning speed. A convergence criterion was proposed to identify the preferred trajectory.

A user study was conducted to learn the drivers’ preferred trajectories of the lane centering control (LCC) system in a simple curve condition without other vehicles. A total of 14 experienced and 15 inexperienced subjects participated in the experiment. The results demonstrate that the OPPLM converges rapidly, within approximately 11 queries on average, and that the driver evaluation scores of the trajectories are consistent with the estimated utility by the learned driver preference model. The OPPLM can quickly and accurately learn the preferences of most subjects.

However, several limitations need to be addressed. The conflict of query answers is common, causing a delay in convergence and inaccurate preference estimation. To ensure adequate exploration, more exploration is necessary for occasional situations. The perception models are not estimated accurately enough when the OPPLM converges because the query selection method and convergence criteria are directly related to UEM only. The trajectory indicators used to build perception models are chosen based on experience, which can be optimized and even adapted to each individual. The estimation method settings in Section 3.4.2 are established based on experience and should be studied further to improve the performance of OPPLM. 

## 7. Patents

A patent is being applied for based on this research.

## Figures and Tables

**Table 1:** The trajectory indicators used to build perception models.

Perception Model	Indicators	Meaning
SPM	Ind_S1	Maximum left lateral offset to lane center
	Ind_S2	Maximum right lateral offset to lane center
	Ind_S3	Minimum time to lane crossing
CPM	Ind_C1	The range of lateral offset to lane center
	Ind_C2	Mean value of speed jerk
	Ind_C3	Mean value of yaw acceleration
EPM	Ind_E1	Minimum speed
	Ind_E2	Maximum speed acceleration
	Ind_E3	Mean inverse time to right lane crossing

**Table 2:** Preference query questionnaire.

Query	Alternatives		
Q1: Which one do you feel safer?	First	Second	Almost same
Q2: Which one do you feel more comfortable?	First	Second	Almost same
Q3: Which one do you think is more efficient?	First	Second	Almost same
Q4: Which one do you prefer?	First	Second	Almost same

**Table 3:** Trajectory evaluation Likert scale.

Item	Scale
I feel very safe.	1 (strongly disagree)–7 (strongly agree)
I feel very comfortable.	1 (strongly disagree)–7 (strongly agree)
I feel very efficient.	1 (strongly disagree)–7 (strongly agree)
I like the way it drives.	1 (strongly disagree)–7 (strongly agree)

**Table 4:** Summary table of GOF of driver preference model of all subjects.

Driver Preference Model	Subjects	Mean	SD
UEM	All	0.85	0.14
	Inexperienced	0.82	0.12
	Experienced	0.88	0.15
SPM	All	0.68	0.18
	Inexperienced	0.69	0.20
	Experienced	0.68	0.17
CPM	All	0.75	0.16
	Inexperienced	0.70	0.15
	Experienced	0.80	0.17
EPM	All	0.64	0.20
	Inexperienced	0.65	0.24
	Experienced	0.64	0.17

**Table 5:** Summary table of SUC of driver preference model of all subjects.

Driver Preference Model	Subjects	Mean	SD
UEM	All	0.74	0.24
	Inexperienced	0.73	0.26
	Experienced	0.74	0.23
SPM	All	0.56	0.41
	Inexperienced	0.62	0.38
	Experienced	0.50	0.47
CPM	All	0.48	0.36
	Inexperienced	0.51	0.28
	Experienced	0.45	0.44
EPM	All	0.11	0.51
	Inexperienced	0.18	0.15
	Experienced	0.05	0.45

**Table 6:** Summary table of evaluation scores of the selected four trajectories according to the estimated utility by each model.

Driver Preference Model	Trajectory	Mean	SD	t ^1^	*p*-Value
UEM	1st	6.48	0.57	-	-
	2nd	5.31	0.85	7.44	0.000
	15th	3.69	1.07	8.60	0.000
	Last	2.93	1.10	3.99	0.000
SPM	1st	6.31	0.85	-	-
	2nd	5.41	1.02	4.77	0.000
	15th	3.69	1.17	7.79	0.000
	Last	3.31	1.54	1.65	0.110
CPM	1st	6.28	0.92	-	-
	2nd	5.14	1.13	5.78	0.000
	15th	4.07	1.39	4.31	0.000
	Last	3.62	1.32	1.47	0.152
EPM	1st	5.24	1.38	-	-
	2nd	5.17	1.26	027	0.787
	15th	4.83	1.28	1.26	0.217
	Last	4.55	1.53	0.87	0.392

^1^ The t-statistic value for the paired-samples test between adjacent trajectory groups.

## Data Availability

Not applicable.
